# Supplementary material for: Characterizing Infectious Disease Mortality in Severe Mental Illness: A Retrospective Matched Cohort Study
Source: Schizophr Bull. 2026 May 9;52(3):sbag067. doi: 10.1093/schbul/sbag067 (PMC13156512; doi:10.1093/schbul/sbag067)
Supplement: sbag067_Supplementary_Material_CleanCopy [file sbag067_supplementary_material_cleancopy.docx]

**Supplementary Material**

**Characterising associations between severe mental illness and infectious disease mortality: A retrospective matched cohort study**

**Lived Experience Commentary**

As someone with lived experience of mental health issues the fact that we finally have robust research that those with severe mental health issues are likely to die and get very ill at a younger age than their peers who do not have severe mental issues is really important. I am on antipsychotic medication, and this led me to have a greater risk of developing diabetes, and I did indeed develop this, and it has had an impact on my overall wellbeing. I wish that this research had been done before and the fact that it has been done is good and it has the potential to positively impact the lives of many people with severe mental health issues.

However good and relevant this research is it cannot improve things without the help and co-operation of policy makers and indeed those who implement policy and those who educate clinicians. My recommendation is that policy makers institute education for all those who are diagnosed with severe mental health issues so that they know the greater risk that they have in comparison with those who do not have severe mental health issues of developing physical health issues and of dying younger. I would suggest they are also educated about healthy lifestyle choices and are given tailored support which should include access to dieticians, physiotherapists and psychologists to try and follow what they have learnt. I would suggest they have yearly physical health checks including blood tests.  I would suggest much more education for clinicians, including the findings of this research, and how to spot the early signs of physical health issues.

Finally, I suggest that further research connect with the Advanced Discovery Pain Platform (<https://apdp.community/>) and its consortium which looks at chronic pain and its causes. Understanding how pain affects mental health, the role of specific comorbidities, and whether the medications patients take increase the risk of serious physical health issues, and even earlier death, will give us crucial insights and inform targeted education for clinicians. Finally, this would also mean that people have better understanding of the illnesses they might get and be more motivated to follow the education and tailored support I suggested earlier.

| **Table S1.** ICD-10 codes used to define infection mortality* | |
| --- | --- |
| **Infection Category** | **ICD-10 code** |
| Any infection | A00-B99, G00, G01, G02, G03, H62.0, H62.1, H67.0, H67.1, J00, J01, J02, J03, J04, J05, J06, J09, J10, J11, J12, J13, J14, J15, J16, J17, J18, J20, J21, J22, L00, L01, L02, L03, L04, L05, L08, M00, M01, N30, N39.0, N45, N70, N71, N72, N73.0, N73.1, N73.2, N73.3, N73.4, N73.5, N74.0, N74.1, N74.2, N74.3, N74.4, N76.4, N77.0, N77.1, O23, O26.4, O85, O86, O98, R57.2, R65.0, R65.1  Most common in SMI group: J18.0, Bronchopneumonia, unspecified (36.3%)  Most common in non-SMI group: J18.0, Bronchopneumonia, unspecified (31.9%) |
| Sepsis | A40-A41, R57.2  Most common in SMI group: A41.9, Sepsis, unspecified (79.2%)  Most common in non-SMI group: A41.9, Sepsis, unspecified (75.6%) |
| Respiratory infections | Most common in SMI group: J18.0, Bronchopneumonia, unspecified (49.8%)  Most common in non-SMI group: J18.0, Bronchopneumonia, unspecified (45.8%) |
| *Influenza* | J09-J11 |
| *Pneumonia* | J12-J18 |
| *Other respiratory* | J00-J06, J20-J22 |
| Gastrointestinal infections | A00-A09  Most common in SMI group: A04.7, Enterocolitis due to Clostridium difficile (76.9%)  Most common in non-SMI group: A04.7, Enterocolitis due to Clostridium difficile (71.4%) |
| Urogenital infections | N10, N11, N13.6, N30, N39.0, N45, N70, N71, N72, N73.0, N73.1, N73.2, N73.3, N73.4, N73.5, N74.0, N74.1, N74.2, N74.3, N74.4, N76.4, N77.0, N77.1  Most common in SMI group: N39.0, Urinary tract infection, site not specified (91.9%)  Most common in non-SMI group: N39.0, Urinary tract infection, site not specified (100.0%) |
| CNS infections | A17, A32.1, A39.0, A81, A83-A89, B00.3, B00.4, B01.0, B01.1, B02.0, B02.1, B02.2, B05.0, B05.1, B06.0, B26.1, B26.2, B58.2, G00-G03  Most common in SMI group: G03.9, Meningitis, unspecified (33.3%)  Most common in non-SMI group: A81.0, Creutzfeldt-Jakob disease (44.4%) |
| Skin infections | A46, B00-B09, L00-L08  Most common in SMI group: L03.9, Cellulitis, unspecified (44.4%)  Most common in non-SMI group: L03.9, Cellulitis, unspecified (54.5%) |
| HIV or hepatitis infections | B15-B19, B20-B24  Most common in SMI group: B18.2, Chronic viral hepatitis C (29.6%)  Most common in non-SMI group: B18.2, Chronic viral hepatitis C (53.3%) |
| Other infections | A00-B99 (excluding all the above-mentioned codes), H62.0, H62.1, H67.0 and H67.1, M00, M01, O23, O26.4, O85, O86, O98, R65.0, R65.1  Most common in SMI group: A49.0, Staphylococcal infection, unspecified site (16.7%)  Most common in non-SMI group: A16.2, Tuberculosis of lung, without mention of bacteriological or histological confirmation (41.2%) |
| * The most common cause of infection-related mortality in those whose primary cause of death was infection is detailed in each category for both SMI and non-SMI group | |

| **Table S2.** All-cause and infection-related mortality by gender | | | | | | |
| --- | --- | --- | --- | --- | --- | --- |
|  | **Male** | | | **Female** | | |
|  | **SMI (N=41096)** | **No SMI (N=41096)** |  | **SMI (N=43398)** | **No SMI (N=43398)** |  |
|  | *Median (IQR) or N(%)* | *Median (IQR) or N(%)* | *P value* | *Median (IQR) or N(%)* | *Median (IQR) or N(%)* | *P value* |
| All-cause mortality | 6855 (16.7) | 4225 (10.3) | <0.001 | 9292 (21.4) | 4872 (11.2) | <0.001 |
| Age at death |  |  | <0.001 |  |  | <0.001 |
| *<45 years* | 1162 (16.9) | 252 (6.0) |  | 447 (4.8) | 147 (3.0) |  |
| *45 to 64 years* | 1821 (26.6) | 741 (17.5) |  | 1316 (14.2) | 476 (9.8) |  |
| *65 to 84 years* | 2801 (40.9) | 2132 (50.5) |  | 3769 (40.6) | 1950 (40.0) |  |
| *≥85 years* | 1071 (15.6) | 1100 (26.0) |  | 3760 (40.5) | 2299 (47.2) |  |
| Infection mortality (primary cause) | 589 (1.4) | 321 (0.8) | <0.001 | 971 (2.2) | 444 (1.0) | <0.001 |
| Age at death |  |  | <0.001 |  |  | <0.001 |
| *<45 years* | 26 (4.4) | 15 (4.7) |  | 24 (2.5) | <5 (<0.01) |  |
| *45 to 64 years* | 115 (19.5) | 35 (10.9) |  | 90 (9.3) | 12 (2.7) |  |
| *65 to 84 years* | 277 (47.0) | 137 (42.7) |  | 407 (41.9) | 132 (29.7) |  |
| *≥85 years* | 171 (29.0) | 134 (41.7) |  | 450 (46.3) | 296 (66.7) |  |
| Infection types |  |  |  |  |  |  |
| *Sepsis* | 16 (0.04) | 24 (0.06) | 0.206 | 37 (0.09) | 21 (0.05) | 0.036 |
| *Respiratory* | 429 (1.04) | 205 (0.5) | <0.001 | 708 (1.6) | 328 (0.8) | <0.001 |
| *Gastrointestinal* | 18 (0.04) | 8 (0.02) | 0.050 | 34 (0.08) | 13 (0.03) | 0.002 |
| *Renal and urogenital* | 78 (0.2) | 41 (0.1) | 0.001 | 145 (0.3) | 63 (0.1) | <0.001 |
| *CNS* | <5 (<0.01) | 7 (0.02) | 0.096 | 10 (0.02) | <5 (<0.01) | 0.021 |
| *Skin* | 14 (0.03) | 11 (0.03) | 0.548 | 13 (0.03) | 11 (0.03) | 0.683 |
| *HIV/hepatitis* | 18 (0.04) | 12 (0.03) | 0.273 | 9 (0.02) | <5 (<0.01) | 0.083 |
| *Other infections* | 14 (0.03) | 13 (0.03) | 0.847 | 16 (0.04) | <5 (<0.01) | 0.007 |
| Infection mortality (primary/secondary cause)* | 1881 (4.6) | 1158 (2.8) | <0.001 | 2875 (6.6) | 1384 (3.2) | <0.001 |
| Age at death |  |  | <0.001 |  |  | <0.001 |
| *<45 years* | 94 (5.0) | 34 (2.9) |  | 63 (2.2) | 20 (1.4) |  |
| *45 to 64 years* | 395 (21.0) | 134 (11.6) |  | 274 (9.5) | 83 (6.0) |  |
| *65 to 84 years* | 931 (49.5) | 603 (52.1) |  | 1262 (43.9) | 496 (35.8) |  |
| *≥85 years* | 461 (24.5) | 387 (33.4) |  | 1276 (44.4) | 785 (56.7) |  |
| Infection types |  |  |  |  |  |  |
| *Sepsis* | 298 (0.7) | 206 (0.5) | <0.001 | 415 (1.0) | 207 (0.5) | <0.001 |
| *Respiratory* | 1507 (3.7) | 903 (2.2) | <0.001 | 2305 (5.3) | 1105 (2.5) | <0.001 |
| *Gastrointestinal* | 32 (0.08) | 20 (0.05) | 0.096 | 62 (0.1) | 24 (0.06) | <0.001 |
| *Renal and urogenital* | 188 (0.5) | 92 (0.2) | <0.001 | 317 (0.7) | 141 (0.3) | <0.001 |
| *CNS* | <5 (<0.01) | 11 (0.03) | 0.032 | 11 (0.03) | <5 (<0.01) | 0.013 |
| *Skin* | 26 (0.06) | 19 (0.05) | 0.297 | 43 (0.1) | 22 (0.05) | 0.009 |
| *HIV/hepatitis* | 71 (0.2) | 24 (0.06) | <0.001 | 24 (0.1) | 6 (0.01) | 0.001 |
| *Other infections* | 15 (0.04) | 24 (0.06) | 0.149 | 17 (0.04) | 12 (0.03) | 0.353 |
| CNS=central nervous system; HIV=human immunodeficiency virus; IQR=interquartile range; SMI=severe mental illness  *More than one infection type may have been recorded as a cause of death | | | | | | |

| **Table S3.** Fully adjusted hazard ratios for the association between interaction terms (e.g. SMI*gender) and risk of death from any infection (primary cause of death) | |
| --- | --- |
|  | **Any infection** |
|  | *aHR (95% CI)* |
| SMI*Gender |  |
| Female | Ref |
| Male | 1.15 (0.97 to 1.38) |
| SMI*Ethnicity |  |
| White | Ref |
| Black | 1.79 (0.93 to 3.45) |
| Asian | 0.98 (0.55 to 1.77) |
| Mixed | 0.68 (0.10 to 4.69) |
| Other | 1.31 (0.73 to 2.36) |
| SMI*IMD |  |
| 1 (Least) | Ref |
| 2 | 0.61 (0.46 to 0.83)* |
| 3 | 0.74 (0.55 to 0.99)* |
| 4 | 0.66 (0.49 to 0.88)* |
| 5 (Most) | 0.59 (0.44 to 0.78)** |
| SMI*SMI diagnosis |  |
| Schizophrenia/Psychosis | Ref |
| Bipolar disorder | 0.58 (0.47 to 0.71)** |
| Both diagnoses | 0.39 (0.27 to 0.56)** |
| *p<0.05, **p<0.001  Models adjusted for ethnicity, neighbourhood deprivation (IMD), BMI, smoking status, and number of long-term conditions, where appropriate | |

| **Table S4.** Fully adjusted hazard ratios for risk of infection‑related mortality among patients with a schizophrenia/psychosis diagnosis and matched controls, including interaction with SMI and stratified analyses | |
| --- | --- |
|  | **Any infection** |
|  | *aHR (95% CI)* |
| *SMI*schizophrenia/psychosis diagnosis* |  |
| Schizophrenia | Ref |
| Schizoaffective disorder | 0.90 (0.46 to 1.74) |
| Other psychotic disorders | 1.84 (1.50 to 2.26)** |
| *Stratified analysis* |  |
| Schizophrenia (N=24,086) | 1.26 (1.06 to 1.50)* |
| Schizoaffective disorder (N=2139) | 0.99 (0.48 to 2.04) |
| Other psychotic disorder (N=35,315) | 2.45 (2.12 to 2.82)** |
| *p<0.05, **p<0.001  Models adjusted for ethnicity, neighbourhood deprivation (IMD), BMI, smoking status, and number of long-term conditions, where appropriate | |

Figure S1. Adjusted survival curve for infection‑related mortality by severe mental illness (SMI) status. Estimates are based on one imputed dataset. Models were adjusted for ethnicity, neighbourhood deprivation, BMI, smoking status, and number of long-term conditions.


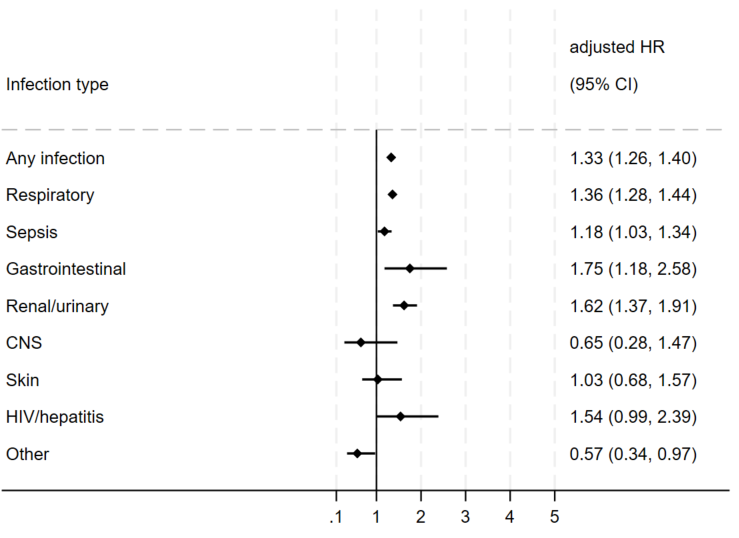

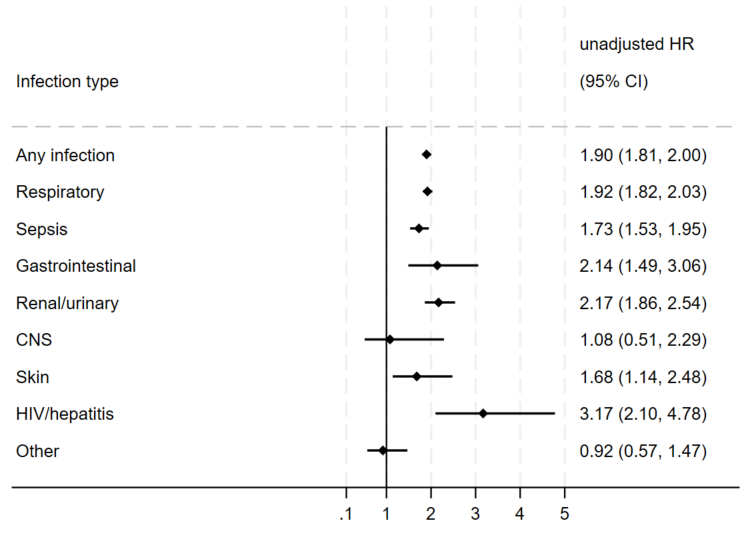


**Figure S2.** Hazard ratios for the association of severe mental illness (SMI) with infection mortality overall and mortality from infection subtypes (as primary and secondary/contributing cause of death). Displayed estimates are from imputed data. Unadjusted models were based on an age-, sex- and GP practice-matched cohort. Fully adjusted models were further adjusted for ethnicity, neighbourhood deprivation, BMI, smoking status, and number of long-term conditions.

**Figure S3.** Fully adjusted hazard ratios for the association of severe mental illness (SMI) with infection mortality overall and mortality from infection subtypes (as primary cause of death) in patients with more than one year of follow-up (N=149,886). Displayed estimates are from imputed data. Fully adjusted models were adjusted for ethnicity, neighbourhood deprivation, BMI, smoking status, and number of long-term conditions.

**Figure S4.** Complete case analysis. Fully adjusted hazard ratios for the association of severe mental illness (SMI) with infection mortality overall and mortality from infection subtypes (as primary cause of death) in patients with more than one year of follow-up (N=102,874). Fully adjusted models were adjusted for age, sex, ethnicity, neighbourhood deprivation, BMI, smoking status, and number of long-term conditions.

**Figure S5.** Fully adjusted odds ratios for the association of severe mental illness (SMI) with infection mortality overall and mortality from infection subtypes (as primary cause of death). Displayed estimates are from imputed data. Fully adjusted models were adjusted for ethnicity, neighbourhood deprivation, BMI, smoking status, and number of long-term conditions.
